# Effects of Chlorogenic Acid on In Vitro Maturation and Vitrification Cryopreservation of Sheep Oocytes

**DOI:** 10.3390/vetsci12010062

**Published:** 2025-01-16

**Authors:** Hong Tao, Yukun Zhao, Qiang Zhang, Xu Li, Guangdong Hu, Yanping Wang, Weibin Zeng

**Affiliations:** College of Animal Science and Technology, Shihezi University, Shihezi 832003, China; taohong@stu.shzu.edu.cn (H.T.); 17699435880@163.com (Y.Z.); ripperzq@outlook.com (Q.Z.); lixu1@stu.shzu.edu.cn (X.L.); guangdonghu@shzu.edu.cn (G.H.)

**Keywords:** sheep, oocyte, CGA, antioxidant

## Abstract

Chlorogenic acid (CGA) has strong antioxidant properties. In order to improve the low maturation rate and poor vitrification freezing effect of sheep oocytes caused by oxidative stress. In this study, oocytes from 200 2–3-year-old Kazakh sheep were collected, and different concentrations of CGA were added to the maturation medium and vitrification freezing solution to study the effects of CGA on the maturation rate, cleavage rate, blastocyst rate, reactive oxygen species (ROS) and glutathione (GSH) levels, mitochondrial membrane potential, and the expression levels of oxidation and apoptosis-related genes in sheep oocytes. The results showed that adding 40 μmol/L CGA to the oocyte in vitro maturation solution significantly increased the maturation rate of oocytes, adding 50 μmol/L CGA to the vitrification cryopreservative solution significantly increased the cleavage and blastocyst rates of mature oocytes activated by parthenogenetic activation after freezing. During in vitro maturation and vitrification freezing in sheep oocytes, CGA significantly reduced the level of ROS and the expression of apoptosis-related genes (Caspase-3 and Bax/Bcl-2), and significantly increased the level of glutathione (GSH), mitochondrial membrane potential, and the expression of antioxidant and anti-apoptosis-related genes (SOD-2 and GPX-3). In addition, CGA significantly increased the expression of the anti-apoptotic gene (AKT) and anti-stress gene (FOXO) during vitrification freezing of sheep oocytes. In conclusion, 40 μmol/L CGA improves the maturation rate of sheep oocytes, and 50 μmol/L CGA improves the quality of parthenogenetic activation embryos after vitrification freezing of mature oocytes in sheep. These results provide a basis for the production of sheep in vitro embryos and the establishment of a germplasm resource bank.

## Simple Summary

Previous studies have shown that mitochondrial dysfunction, cytoplasmic abnormalities, and abnormal embryonic development are linked to excessive intracellular production of reactive oxygen species (ROS). Chlorogenic acid (CGA), a polyphenol widely found in plants, has been shown to have strong antioxidant properties by forming hydrogen radicals to eliminate the activity of free radicals such as hydroxyl radicals and superoxide anions. Thus, this study investigated the effects of CGA on oxidative stress and embryonic development of sheep oocytes by adding different CGA concentrations during in vitro maturation and vitrification freezing. The results showed that CGA effectively alleviates the oxidative stress damage of sheep oocytes, increases their maturation, cleavage, and blastocyst rates after solitary female activation, and promotes embryo development.

## 1. Introduction

In vitro maturation (IVM) of sheep oocytes provides sufficient material for research on embryo engineering. Moreover, vitrification freezing of oocytes safeguards the construction of gene banks for good female animals. However, in vitro reactive oxygen species (ROS)-rich environments cause oxidative stress during oocyte maturation, reducing their quality due to membrane lipid, protein, and DNA damage [1]. Although vitrification freezing reduces the production of ice crystals, it also reduces the ability of oocytes to develop subsequently due to the high cryoprotectant cytotoxicity and oxidative stress [2]. Antioxidants effectively mitigate oocyte damage during in vitro maturation and cryopreservation, thereby increasing their maturation rate and subsequent developmental potential. Specifically, antioxidant proanthocyanidin B1 or green tea extract (epigallocatechin-3-gallate) reduces oocyte apoptosis and oocytes induced by oxidative stress in vitro sow oocyte cultures [3,4]. Melatonin significantly increases the oocyte expansion rate, post-fertilization cleavage, and blastocyst rate in sheep oocyte cultures [5]. Resveratrol has also been shown to be effective in improving the quality of senescent oocytes as well as beneficial for oocyte cryopreservation due to its antioxidant and anti-apoptotic capacity [6]. Additionally, procyanidin B2 significantly reduced the ROS level and improved the oocyte quality in the vitrification freezing solution of female oocytes [7]. Furthermore, it improved the cortical tension of mulberry embryo and blastocyst cells formed by oocyte fertilization after freezing, and the quality of blastocysts [8].

Plants contain chlorogenic acids (CGA), a class of antioxidants that are not cytotoxic [9] and direct wound healing and hepatic injury repair by scavenging ROS and reducing oxidative products and anti-apoptosis [10,11]. The addition of CGA to preservation dilutions of spermatozoa from boars and rams can reduce sperm DNA damage and increase antioxidant parameters, sperm viability, plasma membrane integrity, and mitochondrial membrane potential. Thus, CGA effectively improves the efficiency of artificial insemination [12,13,14,15]. Currently, CGA has only been applied in the study of sow oocytes, where it effectively protects sow oocytes from the DNA damage caused by ROS during in vitro manipulation, CGA significantly improves the maturation rate of sow oocytes and blastocyst rate after parthenogenetic activation during high-temperature fertilization [16]. Moreover, chlorogenic acids also protect oocytes from the effects of heat stress, thereby reducing the frequency of apoptosis and improving embryo quality [17]. However, the role of CGA in the in vitro maturation and vitrification freezing of sheep oocytes is unclear. Thus, this study investigated the effects of different CGA concentrations on in vitro maturation, vitrification freezing, and preservation of sheep oocytes.

## 2. Materials and Methods

### 2.1. Materials

Fetal bovine serum was purchased from Thermo Fisher Scientific (Waltham, MA, USA). MEMA-NEAA (100×), GlutaMAX (100×), BME-EAA (50×) and sodium pyruvate (100 mM) were purchased from Gibco (Waltham, MA, USA). Bovine serum albumin (BSA) was purchased from GPCSCI (Beijing, China). TCM199 basal medium was purchased from Pricella (Wuhan, China). Enhanced mitochondrial membrane potential assay kit (JC-1, C2003SC2003S), ROS assay kit (dichlorofluorescein diacetate, S0035S), TRIzol, hyaluronidase and 6-dimethylaminopurine were purchased from Beyotime (Shanghai, China). Monochlorobimane was purchased from MedChemExpress (Monmouth Junction, NJ, USA). Follicle-stimulating hormone (FSH), β-estradiol, gentamicin, penicillin and CGA were purchased from Solarbio (Beijing, China). NaCl, NaHCO_3_, CaCl_2_-2H_2_O, KH_2_PO_4_, KCl, MgCl_2_-6H_2_O, HiFiScript cDNA Synthesis Kit and 2× UltraSYBR Mixture were purchased from CWBIO (Beijing, China). Dimethyl sulfoxide (DMSO)was purchased from Kulaibo (Beijing, China), and ethylene glycol was purchased from Xinbote (Tianjin, China). Trehalose was purchased from Sigma-Aldrich (St. Louis, MO, USA).

### 2.2. Configuration of CGA Concentration

CGA was dissolved in anhydrous ethanol and diluted to 0, 20, 30, 40, and 50 μmol/L with IVM culture medium and 0, 30, 50, 70, and 90 μmol/L with cryoprotectant solution 2, ensuring that the final concentration of anhydrous ethanol was less than 0.1%.

### 2.3. Configuration of Solutions

Oocyte collection solution: TCM199 basal medium with sodium heparin (0.05 g/L), gentamicin (0.05 g/L) and fetal bovine serum (2%). IVM culture medium: TCM199 basal medium with sodium pyruvate (1 mM), FSH (5 µg/mL), β-estradiol (20 ng/mL), gentamicin (200 µg/mL), Glutamax (2 mM) and fetal bovine serum (10%). Equilibration solution: TCM199 basal medium with fetal bovine serum (20%), ethylene glycol (7.5%) and DMSO (7.5%). Cryoprotectant solution 1: TCM199 basal medium with fetal bovine serum (20%), ethylene glycol (7.5%), DMSO (7.5%) and trehalose (0.25 M). Cryoprotectant Solution 2:TCM199 basal medium with fetal bovine serum (20%), ethylene glycol (15%), DMSO (15%) and trehalose (0.5 M). Thawing Solution 1: TCM199 basal medium with fetal bovine serum (20%), ethylene glycol (7.5%), DMSO (7.5%) and trehalose (0.25 M). Thawing solution 2: TCM199 basal medium with fetal bovine serum (20%) and trehalose (0.13 M). IVC culture solution: Sterile water with NaCl (107.7 mM), NaHCO_3_ (25.07 mM), CaCl_2_-2H_2_O (1.17 mM), KH_2_PO_4_ (1.19 mM), KCl (7.16 mM), MgCl_2_-6H_2_O (0.49 mM), GlutaMax (1 mM), sodium pyruvate (0.4 mM), inositol (2.77 mM), BME-EAA (1%), MEMA-NEAA (1%), BSA (8 mg/mL), gentamicin (25 µg/mL).

### 2.4. Oocyte Collection

A total of 200 ovaries of Kazakh sheep kept in the slaughterhouse of Shihezi city were used in the study. All sheep were kept under natural lighting conditions and diets were formulated based on the nutritional requirements of ewes, and mineral salts were provided in lick bricks. The sheep ovaries, collected from an abattoir in Shihezi, Xinjiang, China, were transported to the laboratory in sterile saline containing 100 IU/mL penicillin within 2 h. The ovaries were placed into the oocyte collection solution and the scalpel incised the follicles to allow the efflux of the oocyte mound–oocyte complex. Oocytes with uniform cytoplasm and encapsulated by three or more layers of granulosa cells were collected, and the collected oocytes were washed twice in the petri dish for use. Under the somatoscopic microscope, the oocytes were transferred to 500 μL of IVM culture medium containing different CGA concentrations and cultured in a 5% CO_2_ incubator at 38.5 °C for 24 h. The effect of CGA on the maturation of sheep oocytes in vitro during IVM was observed.

### 2.5. Vitrification Freezing and Resuscitation of Mature Oocytes

Mature oocytes were equilibrated in equilibrium solution for 1–3 min and then treated in cryoprotectant solution 1 for about 1 min. The oocytes were then treated for 30 s with cryoprotectant solution 2 containing different concentrations of CGA. Finally, the tubes were loaded in five segments using ordinary wheat tubes and inhaled in 0.5 cm alginate lengths at a concentration of 0.5 M, 0.5 cm of air, 1 cm of cryoprotectant solution 2 containing oocytes, 0.5 cm of air, and 0.5 cm of a 0.5 M trehalose. The tubes were sealed and stored in liquid nitrogen.

The oocytes were frozen for 2 weeks, incubated in air for 10 s, and in a water bath at 37 °C for 15 s. The ends were cut off, and the solution and oocytes were blown into thawing solution 1 with a pipette gun for 5 min. Next, oocytes were transferred into thawing solution 2 and washed twice. Finally, oocytes were transferred into a pre-warmed cell culture medium and incubated for 1–2 h for subsequent assays.

### 2.6. Parthenogenetic Activation and Embryo Culture

Four-well plates were filled with 500 μL of IVM medium and 0.1% hyaluronidase per well. The resuscitated mature sheep oocytes were transferred to the culture medium and repeatedly and gently blown using the pipette gun to separate the granulosa cells of the oocyte mound. The oocytes were transferred to IVC culture medium containing 7% ethanol for 7 min [18], IVC culture solution was washed three times. Next, they were transferred to the culture containing 2 mM 6-dimethylaminopurine for 4 h, then to the IVC culture solution for 48 h for development. The oocyte cleavage and blastocyst rates were calculated on the 7th day.

### 2.7. Calculation of the Oocyte Maturation, Cleavage, and Blastocyst Rates

Observed under the microscope: the first polar body was discharged as a sign of oocyte maturation, the occurrence of mitosis as a marker of oocyte cleavage, emergence of the blastocyst cavity as a marker of blastocyst formation.

Oocyte maturation rate = number of mature oocytes/total number of oocytes × 100%

Cleavage rate = number of cleaved cells/number of parthenogenetic activation cells at 48 h × 100%

Blastocyst rate = number of blastocysts/number of cleavage × 100%

### 2.8. Detection of ROS and GSH Levels

After 24 h of in vitro maturation culture, about 15 oocytes each were placed in 100 μL of dichlorofluorescein diacetate and monochlorobimane droplets, and incubated for 20 min at 38.5 °C. The incubated oocytes were washed three times with the culture medium. Finally, the samples were photographed under a fluorescence inverted microscope. In the presence of ROS, dichlorofluorescein diacetate was oxidized to produce green fluorescence, and the fluorescence intensity was proportional to the intracellular ROS level; in the presence of GSH, Monochlorobimane produced blue fluorescence, and the fluorescence intensity was proportional to the intracellular GSH level.

### 2.9. Detection of Mitochondrial Membrane Potentials

After 24 h of in vitro maturation culture, about 20 oocytes were placed in 50 μL of cell culture medium, to which 50 μL of JC-1 staining solution was added. The mixture was incubated for 20 min at 38.5 °C; the oocytes were washed three times with buffer, photographed, and analyzed under the transmission electron microscope. JC-1 monomer at low mitochondrial membrane potential generates green fluorescence, and the j-aggregate formed by JC-1 aggregates at high mitochondrial membrane potential generates red fluorescence. The high potential red fluorescence/low potential green fluorescence is the mitochondrial membrane potential.

### 2.10. Real-Time Quantitative RT-PCR

Primer5.0 software was applied for primer design (see Table 1) according to the sheep gene sequences in NCBI. About 50 mature oocytes were randomly selected from each group for mRNA extraction using the TRIZOL method. The mRNA was reverse transcribed into cDNA templates according to the reverse transcription kit instructions, The reaction system (20 μL): 4 μL of dNTP Mix, 2 μL of Primer Mix, 1 μL of RNA Template, 4 μL of 5× RT Buffer, 2 μL of DTT, and 1 μL of HiFiScript6 μL of RNase-Free Water, the reaction procedure: 42 °C for 15 min, followed by 85 °C for 5 min. Gene expression was detected using the LightCycler 96 (Roche Diagnostics, Basel, Switzerland) with GAPDH as an internal reference, the reaction system (20 μL): 2 μL of cDNA template, 10 μL of SYBR mix, 0.8 μL of forward and reverse primers, and 7.2 μL of ddH_2_O, the reaction procedure: pre-denaturation at 95 °C for 30 s, followed by 95 °C for 10 s, 60 °C for 20 s and 72 °C for 32 s, for 45 cycles.

### 2.11. Statistical Analysis

Each group of experiments was performed thrice. Mean fluorescence intensity was measured using ImageJ 1.53 software (Mean fluorescence intensity = sum of fluorescence intensity in the area/area of the area). The RT-qPCR results were processed using the 2^−ΔΔCt^ method. The data were analyzed by one-way ANOVA with Duncan’s method post hoc test using SPSS 20.0 software. *p* < 0.05 indicates significant differences, and *p* < 0.01 indicates highly significant differences.

## 3. Results

### 3.1. Effect of CGA on the Maturation of Sheep Oocytes In Vitro

**Figure 1:**
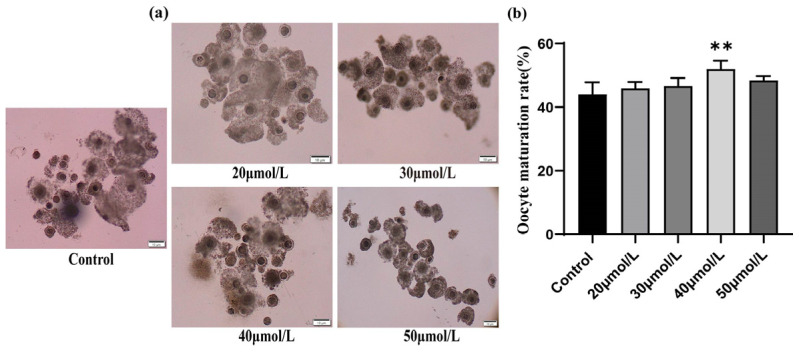
Effects of different CGA concentrations on ovine oocyte maturation in vitro. (**a**) Biological microscopy was used to observe oocyte maturation under different CGA concentrations. (**b**) The maturation rate of ovine oocytes under different CGA concentrations. The total number of oocytes at each concentration was 180, 172, 179, 190, and 195, respectively. ** indicates *p* < 0.01. The results are expressed as mean ± standard deviation. R = 3.

The maturation rate was used as the criterion to evaluate the effect of different CGA concentrations (0, 20, 30, 40, and 50 μmol/L) on oocyte maturation in vitro. Oocyte growth was better in the 40 μmol/L CGA-treated than in the 0 μmol/L CGA-treated group (Figure 1a). The oocyte maturation rate was positively correlated with CGA concentration and was significantly higher in the 40 μmol/L (51.99 ± 2.57%) than in the 0 μmol/L treatment group (44.08 ± 3.74%, *p* < 0.01, Figure 1b). Therefore, 40 μmol/L CGA was chosen for subsequent experiments.

### 3.2. Effect of CGA on ROS, GSH, and the Mitochondrial Membrane Potential in Oocytes

**Figure 2:**
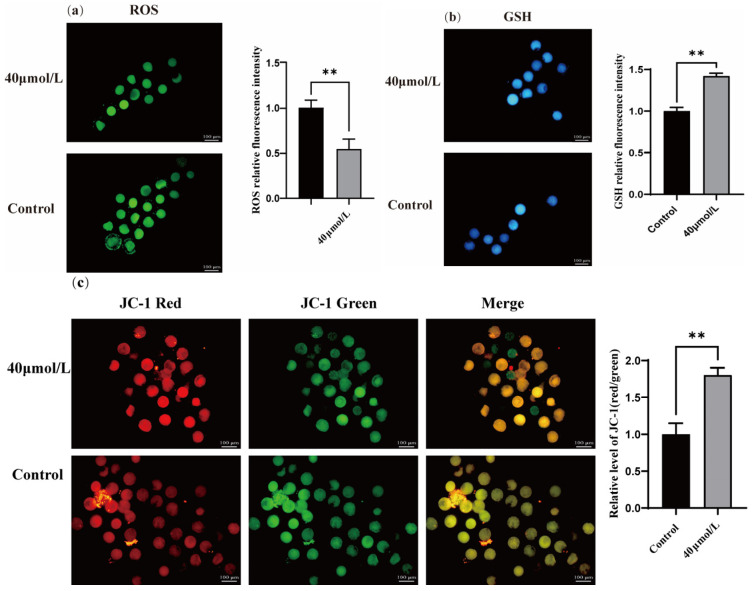
Effects of CGA on oocyte ROS, GSH, and mitochondrial membrane potential. (**a**) Dichlorofluorescein diacetate staining was used to detect ROS levels in mature ovine oocytes. (**b**) Monochlorobimane staining was used to detect GSH levels in mature ovine oocytes. (**c**) JC-1 staining detected changes in the mitochondrial membrane potential of mature ovine oocytes. ** indicates *p* < 0.01. The results are expressed as mean ± standard deviation. R = 3.

Treatment with CGA significantly increased the GSH (Figure 2b, *p* < 0.01) and mitochondrial membrane potential (Figure 2c, *p* < 0.01). In contrast, CGA treatment significantly reduced the ROS level in oocytes (Figure 2a, *p* < 0.01).

### 3.3. Effect of CGA on Antioxidant and Apoptosis-Related Genes in Sheep Oocytes

**Figure 3:**
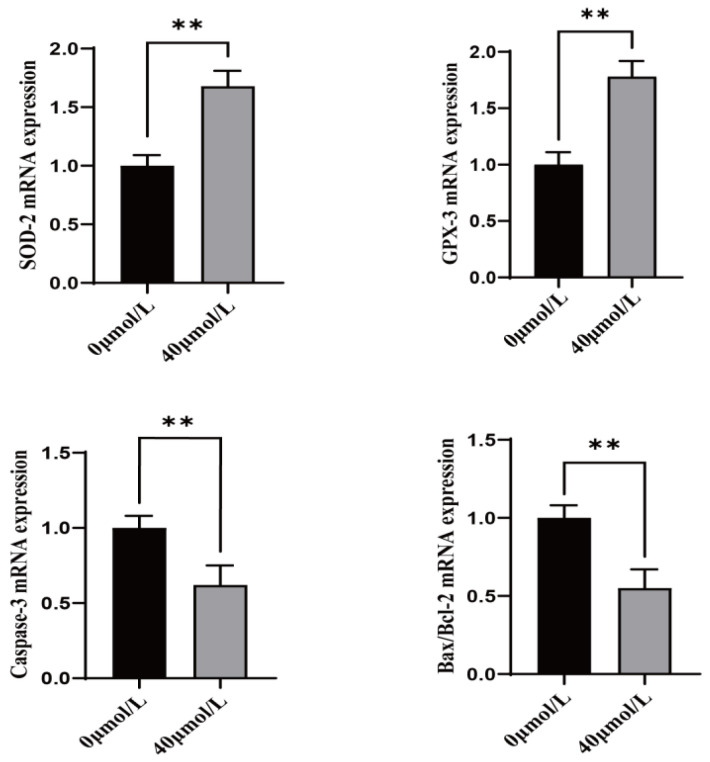
Effects of CGA on antioxidant genes SOD-2 and GPX-3 and, apoptosis-related genes Caspase-3 and Bax/Bcl-2 ratio (Bax is the pro-apoptotic gene, Bcl-2 is the anti-apoptotic gene, and its ratio represents the level of apoptosis) in sheep oocytes. ** indicates *p* < 0.01. The results are expressed as mean ± standard deviation. R = 3.

Treatment with CGA significantly increased the expression of antioxidant genes (SOD-2 and GPX-3) (Figure 3, *p* < 0.01) and significantly decreased the expression of apoptosis marker genes (Caspase-3 and Bax/Bcl-2) (Figure 3, *p* < 0.01) in oocytes.

### 3.4. Effect of CGA on Vitrification Freezing of Mature Sheep Oocytes

**Figure 4:**
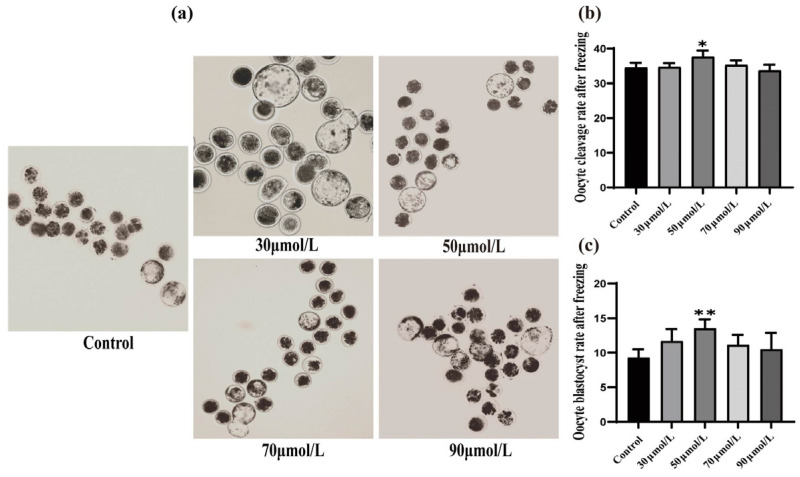
Effects of different CGA concentrations on the cleavage and blastocyst rates of oocytes during vitrification and freezing. (**a**) The effects of different CGA concentrations on blastocyst growth, observed by biological microscopy. (**b**) The ovine oocyte cleavage rate was calculated after 48 h in each CGA treatment group. (**c**) On day 7, ovine oocyte blastocyst rates were calculated for each CGA treatment group. The total number of oocytes at 152, 147, 162, 176 and 158, respectively. The total number of mature oocytes entering parthenogenesis activation at different concentrations was 79, 76, 83, 91 and 82, respectively. * indicates *p* < 0.05. ** indicates *p* < 0.01. The results are expressed as mean ± standard deviation. R = 3.

The cleavage rate and blastocyst rate were used as the criterion to evaluate the effect of different CGA concentrations (0, 30, 50, 70, and 90 μmol/L) on oocyte vitrification cryopreservation. The cleavage rate (34.54%, Figure 4b, *p* < 0.05) blastocyst rate (13.53%, Figure 4c, *p* < 0.01) of the 50 μmol/L CGA-treated group was significantly higher than that of the control.

### 3.5. Effect of CGA on ROS, GSH, and Mitochondrial Membrane Potential During Vitrification Freezing of Sheep Oocytes

**Figure 5:**
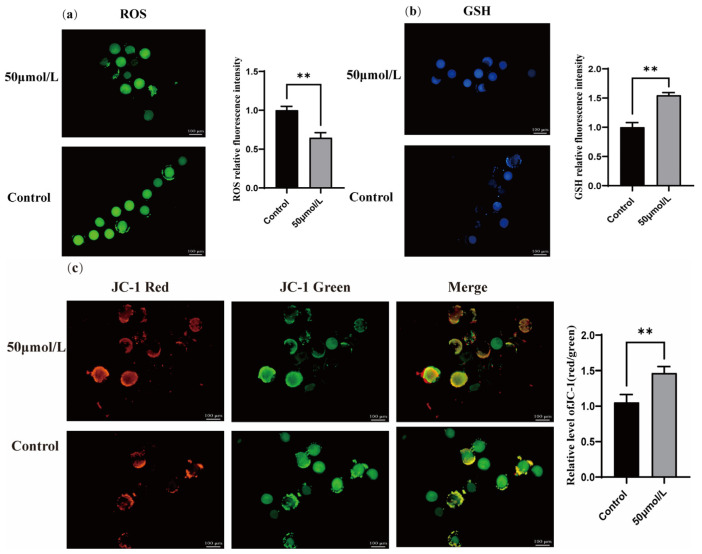
Effects of CGA on ROS, GSH, and mitochondrial membrane potential during oocyte freezing. (**a**) DCFH-DA staining was used to detect ROS levels in oocytes after freezing. (**b**) Momochlorobi-mane staining was used to detect GSH levels in oocytes after freezing. (**c**) Changes in the mitochondrial membrane potential of ovine oocytes after freezing with JC-1 staining. ** indicates *p* <0.01. The results are expressed as mean ± standard deviation. R = 3.

Treatment with CGA significantly increased the GSH level (Figure 5b, *p* < 0.01) and the mitochondrial membrane potential (Figure 5c, *p* < 0.01). In contrast, CGA treatment significantly reduced the ROS levels in oocytes (Figure 5a, *p* < 0.01).

### 3.6. Effect of CGA on Antioxidant, Apoptosis, and Anti-Stress Related Genes During Freezing of Sheep Oocytes

**Figure 6:**
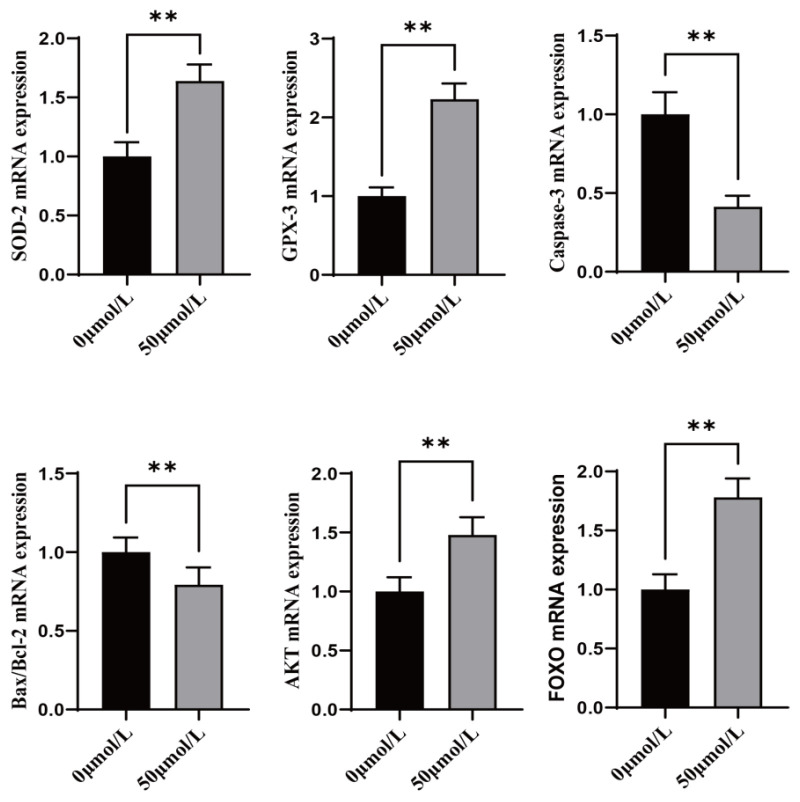
Effects of CGA on genes related to antioxidant SOD-2 and GPX-3, apoptosis Caspase-3 and Bax/Bcl-2 (Bax is the pro-apoptotic gene, Bcl-2 is the anti-apoptotic gene, and its ratio represents the level of apoptosis), anti-apoptosis AKT, and anti-stress FOXO during oocyte vitrification and freezing. ** indicates *p* < 0.01. The results are expressed as mean ± standard deviation. R = 3.

Treatment with CGA significantly increased the expression of antioxidant genes (SOD-2 and GPX-3), anti-stress genes (FOXO), and anti-apoptosis genes (AKT) in oocytes (Figure 6, *p* < 0.01). However, CGA treatment significantly decreased apoptosis marker genes (Caspase-3 and Bax/Bcl-2).

## 4. Discussion

Reactive oxygen species are strongly oxidizing. Excessive ROS accumulation beyond the normal oocyte antioxidant capacity causes intracellular mitochondrial dysfunction and cytoplasmic abnormality [19,20]. Moreover, antioxidant addition can reduce the oxidative damage to oocytes by decreasing the ROS concentration [21]. Chlorogenic acid, a polyphenolic substance found in honeysuckle, dulcimer, and other traditional Chinese herbs, has higher contents of polyphenols. The polyphenols can eliminate the activity of free radicals such as hydroxyl radicals and superoxide anion and have concentration-dependent strong antioxidant properties [22]. In the study, adding 40 μmol/L CGA to the IVM culture medium significantly increased the in vitro maturation rate of sheep oocytes (51.99%) compared to the control group (44.08%).

Furthermore, resuscitation by adding 50 μmol/L CGA in vitrified cryoprotectants significantly increased the oocyte cleavage rate (37.67%) and blastocyst rate (13.53%) compared to that of the control group (34.54%, 9.25%, respectively). Previous studies showed that adding the antioxidants eugenol, chenopodium, or chrysin can alleviate oxidative damage in bovine oocytes. The alleviation involves maintaining the mitochondrial membrane potential and decreasing the endoplasmic reticulum stress, thus improving the cleavage rate and embryo quality of bovine oocytes [23,24,25]. Rhodiola rosea alleviates the oxidative stress induced by mouse oocyte senescence by decreasing ROS levels [26]. Melatonin also alleviates mitochondrial dysfunction and reduces the rate of meiotic abnormality in postovulatory-aged mouse oocytes via a SIRT1-MnSOD-dependent pathway [27]. Additionally, applying N-acetylcysteine during vitrification freezing of sheep GV-stage oocytes improved freezing efficiency [28].

Glutathione has a strong protective effect on cells against oxidative stress and is key in oocyte maturation, meiosis, spindle formation, and maintenance [29]. Thus, GSH depletion in the antioxidant defense system of oocytes exacerbates the oxidative damage caused by ROS accumulation [30]. The addition of antioxidants can effectively increase GSH levels in oocytes [31]. In this study, adding CGA to the oocyte in vitro maturation culture medium and vitrification cryopreservation solution significantly decreased the ROS level in sheep. Moreover, CGA significantly increased the GSH level, implying that CGA can effectively improve oxidative stress during oocyte in vitro maturation and vitrification freezing. Additionally, the distribution, structure, and activity of oocyte mitochondria can influence cellular function.

Mitochondrial activity is adaptive and responsive to the oocyte maturation process at the whole-cell and local levels [32]. Nonetheless, energy production by the mitochondria in the cell is accompanied by copious ROS generation [33]. Studies have demonstrated that decreasing the mitochondrial membrane potential in oocytes causes abnormal oocyte development [34]. Thus, changes in the mitochondrial membrane potential can be a reliable indicator for assessing the quality of oocytes. In this study, adding CGA to the in vitro maturation culture and vitrification cryopreservation solutions of sheep oocytes significantly increased their mitochondrial membrane potentials. Therefore, CGA could restore mitochondrial function and improve the quality of oocytes.

Superoxide dismutase (SOD) and glutathione peroxidase (GPX-3) can inhibit endogenous apoptosis by decreasing the content of ROS [35,36]. In this experiment, adding CGA to the in vitro maturation culture fluid and vitrification cryopreservation fluid of sheep oocytes revealed that CGA significantly increased the expression of SOD-2 and GPX-3. Therefore, CGA probably promotes the expression of antioxidant-related genes to alleviate oxidative stress in oocytes. As an important gene in apoptosis, Caspase-3 on the PI3K-AKT pathway, when the activation of AKT, it is able to inhibit the activity of Caspase-3 under certain conditions, thereby reducing apoptosis [37]. Similarly, the anti-apoptotic gene BCL-2 and the apoptotic gene BAX are antagonistic genes whose ratio of expression can determine whether the fate of cells is apoptosis or proliferation [38]. In this study, the addition of CGA to the oocyte in vitro maturation culture medium and vitrification cryoprotective solution significantly reduced the expression of Caspase-3 and the ratio of BCL-2/BAX. Interestingly, the changes in BCL-2 and BAX expression were almost synchronous, but the BCL-2 gene was up-regulated by a greater fold. These results indicate that CGA-treated oocytes continue growing and developing rather than undergoing apoptosis. Besides the relevant oxidative apoptosis genes during vitrification freezing, this study also examined the FOXO gene [39], which is considered to maintain cellular and organismal homeostasis by regulating stress response pathways. Treatment with CGA significantly up-regulated expression of the FOXO gene. Therefore, CGA can mitigate oxidative stress damage in sheep oocytes during in vitro maturation and also has a protective effect on the developmental potential of oocytes after freezing.

## 5. Conclusions

Altogether, these results showed that the addition of 40% CGA to the IVM culture solution and 50% CGA to the vitrification freezing solution can increase their maturation rate as well as the cleavage and blastocyst rates after freezing, respectively. These findings provide a basis for in vitro embryo production and oocyte genetic material preservation in sheep.

## Figures and Tables

**Table 1:** qRT-PCR primer sequences.

Gene Name	GenBank Accession	Sequence (5′–3′)	Length (bp)
SOD-2	NM_001280703.1	F:AGGGAGATAAAGTCGTCGTA	165
		R:ACAGAGGATTAAAGTGAGGG	
GPX3	XM_015096153.3	F:CCATTCGGTCTGGTCATT	176
		R:CCCGTTCACATCGCCTTT	
BCL-2	XM_012103831.5	F:GCCGAGTGAGCAGGAAGAC	312
		R:GTTAGCCAGTGCTTGCTGAGA	
BAX	XM_027978592.3	F:CCTGGGATCTTGAAACTCTCCTT	148
		R:CTGAGCCAGGCTGAAATCAAAA	
Caspase-3	XM_060406953.1	F:AAGTTTCTTCAGAGGGGACTGTTGC	177
		R:GCCATGTCGTCCTCAGAACCAC	
AKT	XM_027956972.3	F:AGTACATCAAGACCTGGCGG	216
		R:GAGAAGTTGTTGAGGGGCGA	
FOXO	XM_060419173.1	F:AGTACCATTAGCGGGAGGCT	110
		R: GTAGAAGCCATCTTTGCGGC	
GAPDH	XM_060411593.1	F: CGGCACAGTCAAGGCAGAGAAC	114
		R: CACGTACTAGCACCAGCATCAC	

## Data Availability

The datasets presented in this study can be found in the article.
